# Effectiveness of care in acute dizziness presentations

**DOI:** 10.1007/s00405-019-05470-0

**Published:** 2019-05-16

**Authors:** Mikael Granberg Sandlund, Anna Diamant, Gabriel Granåsen, Jonatan Salzer

**Affiliations:** 1 Department of Pharmacology and Clinical Neuroscience, Section of Neurology, Umeå University, SE-901 87 Umeå, Sweden; 2 Department of Public Health and Clinical Medicine, Epidemiology and Global Health Unit, Umeå University, Umeå, Sweden

**Keywords:** Dizziness, Vertigo, Health economics, Management algorithms, Effectiveness of care

## Abstract

**Purpose:**

This study aims to evaluate whether a management algorithm has improved the effectiveness of care for dizzy patients at Umeå University Hospital.

**Methods:**

This was an interventional study using medical records to collect data for acute dizziness presentations before (period 1, 2012–2014) and after (period 2, 2016–2017) the implementation of a management algorithm. Outcomes were changes in a set of pre-defined effectiveness markers and health economic effects.

**Results:**

Total *n* = 2126 and *n* = 1487 acute dizziness presentations were identified in period 1 and 2, respectively. Baseline characteristics were similar. The proportion of patients undergoing Dix–Hallpike testing increased, 20.8% [95% confidence interval (CI) 18.8–23.0%] vs. 37.7% (95% CI 35.2–40.2%), as did BPPV diagnoses, 7.6% (95% CI 6.6–8.8%) vs. 15.3% (95% CI 13.6–17.3%). Hospitalization became less common, 61.5% (95% CI 59.4–63.6%) vs. 47.6% (95% CI 45.1–50.2%). The proportion undergoing any neuroradiological investigation decreased, 44.8% (95% CI 42.7–47.0%) vs. 36.3% (95% CI 33.8–38.7%) with a shift from CT to MRI, with unchanged sensitivity for diagnosing cerebrovascular causes. The average cost for the care of one dizzy patient decreased from $2561 during period 1 to $1808 during period 2.

**Conclusions:**

This study shows that the implementation of a management algorithm for dizzy patients was associated with improved effectiveness of care.

## Introduction

Dizziness and vertigo, hereafter termed “dizziness” for practical reasons, [1] are common symptoms presented by patients at emergency departments (EDs) [2]. Several studies have shown that there is room for improvement in the care of dizzy patients. A large proportion of dizzy patients remain undiagnosed [3, 4], patients often revisit the ED several times for the same complaints [4] and the use of neuroradiological investigations is increasing, inflating health-care spending [5, 6]. Fear of missed strokes among dizzy patients may be one of the factors behind this trend, although peripheral vestibular, non-neurological cardiovascular, respiratory, metabolic and psychiatric causes are more common causes of dizziness than stroke and TIA according to most reports [3, 4, 6–9].

A retrospective study at Umeå University Hospital covering 2012–14 showed that more than half of ED patients with dizziness remained undiagnosed despite an unusually high degree ( > 60%) of hospitalization; and that few underwent positional testing for the most common peripheral vestibular causes of dizziness [4]. These findings suggested that this hospital had much to gain from measures aiming at increasing the effectiveness of care for dizzy patients. Thus, in the spring of 2015, a management algorithm (“Project Imbalance”) for the care for dizzy patients at Umeå University Hospital was launched. The objectives of the present study were to investigate, compare and report any changes in the effectiveness of care, as defined below, for dizzy patients at Umeå University Hospital after the implementation of this management algorithm for dizziness.

## Materials and methods

### Study population and study design

This was an interventional study comparing effectiveness of care for dizzy patients before and after the implementation of a management algorithm. All patient visits to the Umeå University Hospital ED, the only hospital serving this catchment area, due to dizziness during 2012–01-01 to 2014–12-31 (period 1) vs. 2016–01-01 to 2017–12-31 (period 2) were analyzed. Patients were triaged by ED nurses who identified “dizziness” as the main reason for contact, including vertigo and giddiness (Swedish: “yrsel/svindel”) using the standardized RETTS® (Rapid Emergency Triage and Treatment System) triage system [10]. Exclusion criteria were age below 18 years, classified or missing charts, patients referred to primary care without seeing an ED physician, and if there was no mention of dizziness or similar symptom(s) in the charts.

### Data extraction

Data abstracted from medical charts were age, gender, risk factors for stroke, antiplatelet and anticoagulant medications, past cardiovascular illnesses, diagnostic examinations, associated symptoms and clinical neurological findings, in-patient care data, otolaryngology department consultation, dates, discharge diagnoses and any stroke diagnosis within 90 days after the index ED visit. Data regarding Dix–Hallpike testing were mistakenly only collected for two of the years, 2012–13, during period 1 which is why 2014 was excluded from the comparisons of Dix–Hallpike testing frequencies. Data abstraction for period 1 was performed as previously detailed [4]. For period 2, data were collected by a medical student (MGS) and uncertainties were discussed together with the senior author (JS), a neurologist, who also validated all cerebrovascular diagnoses and acute vestibular syndromes (AVS), including all cases with suspected stroke or AVS, according to study diagnostic criteria (below). Missing data were considered not present. The project received ethical committee approval.

### Definitions and diagnoses

AVS was defined as new (since ≤ 72 h) ongoing and continuous dizziness fulfilling at least two of the following criteria: (a) nystagmus (b) nausea or vomiting (c) gait or balance disturbance during physical examination, and (d) discomfort with head movement. Focal neurological deficits were defined as any sensory or motor deficit(s) including language signs (dysarthria or aphasia), disrupted function of cranial nerves, or neglect. The definition of ataxia was dysmetria during heel–shin- or finger–nose-test, inability to sit unaided, or gait ataxia. Stroke and TIA were defined as sudden onset focal neurological deficit (not better explained by other factors) or acute ischemia or non-traumatic intracerebral or subarachnoidal bleeding seen on computed tomography (CT) or magnetic resonance imaging (MRI). A diagnosis of transient ischemic attack (TIA) required regress of symptoms within 24 h. A neuroradiological investigation was considered “diagnostic” when revealing findings with a plausible association with the presenting symptoms.

Similar to period 1 [4], ICD-10 diagnoses for period 2 were left unchanged with the following pre-planned exceptions: (a) patients who had received symptom diagnoses (e.g. R42.9) but met the criteria for cerebrovascular disease were assigned a cerebrovascular disease diagnosis (*n* = 3), and patients who had received a cerebrovascular disease diagnosis but did not meet the criteria for such a diagnosis were assigned a symptom diagnosis (*n* = 2); (b) patients who had received a symptom diagnosis but where the physician clearly stated a medical diagnosis in text were assigned a medical diagnosis (*n* = 12), and patients without a benign paroxysmal positional vertigo (BPPV) diagnosis but where findings during Dix–Hallpike testing suggested BPPV, i.e. nystagmus or vertigo during testing with resolution of symptoms after a repositioning maneuver, were assigned a BPPV diagnosis (*n* = 11), and (c) patients in whom ear–nose–throat (ENT) physician consultation suggested a peripheral vestibular diagnosis rather than an earlier unspecific symptom diagnosis were assigned a peripheral vestibular diagnosis (*n* = 36). Validation and re-classification of diagnoses outside the cerebrovascular and peripheral vestibular ones were not deemed feasible.

### The management algorithm

Project imbalance has been running since April 27th, 2015 and has included the implementation of a management algorithm at Umeå University Hospital to identify dizzy patients at high risk of stroke. This comprehensive vertigo/dizziness management algorithm consists of guidelines for uniform identification and management of cases with an AVS at several different levels of care, i.e. in the nurses’ triage, at the ED physicians’ evaluation and during in-hospital care. Physicians and nurses at the ED were instructed during nine non-mandatory mixed theoretical/practical training sessions by the senior author between May 2014 and September 2018. The staff were trained to carefully evaluate each case with positional testing for BPPV and to use the appropriate repositioning maneuver, if applicable; to screen for AVS according to the AVS criteria; and to perform routine neurological investigation. In case of AVS, a subset of dizzy patients, the instructions were to admit to the stroke ward and perform an MRI with diffusion-weighted images ≥ 24 h from ictus, as these patients have been shown to have an increased risk for cerebrovascular disease compared with non-AVS patients, and as MRI sensitivity for small posterior fossa ischemias may increase over the first day(s) [4, 11, 12]. The patients with AVS also performed a video head impulse test and nystagmus recording session, and were evaluated by an ENT physician and a physiotherapist. Non-AVS patients without other signs of dangerous causes were usually discharged directly from the ED (although the algorithm did not specify how to manage these patients).

### Data analyses and statistics

Data for the effectiveness of care were compared between the pre-management algorithm implementation period (2012–01-01 until 2014–12-31, period 1), and the post-management algorithm implementation period (2016–01-01 until 2017–12-31, period 2). The year 2015 was omitted as the algorithm had not yet been fully implemented. The following six pre-defined markers of effectiveness of care constituted the main outcomes*: (*a) the proportion of undiagnosed patient visits (b) vestibular testing (i.e. the proportions of patients undergoing Dix–Hallpike testing, and the proportion seeing an ENT physician) (c) admittance (i.e. the proportion of hospitalized patients and length-of-stay) (d) the use of neuroradiological investigations (e) the proportion of returning dizzy patients and patients suffering a new onset stroke within 90 days and (f) the cost of care. Patients were followed during an extended 90 days after each time period regarding new onset stroke diagnoses. It was not possible to extend the observation time in a similar manner regarding returning dizzy patients which is why index visits for this variable were not considered during the last 90 days of each period. To control for possible organizational changes influencing rates of hospitalization and length-of-stay we also investigated the numbers of available hospital beds and the occupancy, as well as the mean and median lengths-of-stay for a control group not expected to change due to the effects of the management algorithm, i.e. headache (R51.9, G43.x and G44.x) patients, during the study duration. Statistical analyses were performed in IBM SPSS Statistics version 24 (IBM SPSS Statistics for Windows, Armonk, NY: IBM Corp., released 2016), and WinPepi version 11.17. The independent samples *t* test was used to test equality in mean values. When not applicable, the Mann–Whitney *U* test was used and this was noted in the text. We assumed a Poisson distribution when calculating 95% confidence intervals (CIs) for incidences. Cost estimations were based on the local economical governance groups’ pricing lists for 2017, including all running costs for the departments in question, delivered to us as a per unit estimate. For these calculations, we considered the costs for ED visits (including laboratory diagnostics), in-patient care (excluding laboratory diagnostics), and neuroradiological investigations. Each started day counted as one whole day of care (i.e. one over-night stay yielded two days of in-hospital care). To approximate the costs for CT scans, as data on date and time and CT-angiography were not collected for all visits, we retrospectively collected these data on every 20th investigation during the 5 years of study duration and weighted the costs based on this. The 8th of September 2018 exchange rate [$1 = 9.78 Swedish crowns (SEK)] was used to convert currencies. A *p* value below 0.05 was considered to indicate statistical significance.

## Results

### Study population and characteristics

Detailed data for the *n* = 2126 dizziness visits in period 1 were published [4]. During period 2, *n* = 1716 patients were triaged by the nurses at the ED as “yrsel/svindel” (i.e. dizziness, including vertigo and giddiness). After the exclusion of *n* = 229 visits due to 1) reason for visit not dizziness (*n* = 183), 2) missing documentation (*n* = 39) and 3) confidential charts (*n* = 7), the final cohort for period 2 consisted of *n* = 1487 patient visits. Baseline characteristics were similar between the two periods, with a slight female preponderance, and a mean age of just above 60 years. The two cohorts were also comparable regarding cardiovascular comorbidities, however, slightly fewer during period 2 had hyperlipidemia, congestive heart failure and previous stroke (Table 1). The population-based yearly incidences of dizziness during periods 1 and 2 were similar, 478 (458–498) vs. 483 (459–508) per 100,000 inhabitants.

**Table 1:** Baseline characteristics for emergency department dizziness visits at Umeå University Hospital comparing period 1 (2012–2014) with period 2 (2016–2017)

Variable	Period 1 (*n* = 2126)	Period 2 (*n* = 1487)	Difference (95% CI)
Age, years, mean (SD)	63.8 (18.9)	62.6 (19.4)	– 1.2 (– 2.5 to 0.1)
Female* n* (% of total)	1209 (56.9)	865 (58.2)	1.3% (– 2.0 to 4.6%)
Cardiovascular comorbidities, *n* (% of total)			
Hypertension	1099 (51.7)	729 (49.0)	– 2.7% (– 6.0 to 0.6%)
Diabetes	257 (12.1)	173 (11.6)	– 0.5% (– 2.6 to 1.7%)
Hyperlipidemia	437 (20.6)	234 (15.7)	– 4.8% (– 7.3 to – 2.3%)
Congestive heart failure	157 (7.4)	66 (4.4)	– 3.0% (– 4.5 to – 1.4%)
Atrial fibrillation	290 (13.6)	177 (11.9)	– 1.7% (– 3.9 to 0.5%)
Previous TIA	99 (4.7)	64 (4.3)	– 0.4% (– 1.7 to 1.1%)
Previous stroke	231 (10.9)	124 (8.3)	– 2.5% (– 4.4 to – 0.6%)
Clinical characteristics (% of total)			
AVS	408 (19.2)	183 (12.3)	– 6.9% (– 9.2 to – 4.5%)
Nystagmus	223 (10.5)	144 (9.7)	– 0.8% (– 2.8 to 1.2%)
AVS with nystagmus (% within AVS)	160 (39.2)	112 (61.2)	22.0% (13.3–30.2%)
Focal neurological deficit^a^ (excl. Ataxia)	76 (3.6)	44 (3.0)	0.6% (– 0.6 to 1.8%)
Transient focal deficit^a^	64 (3.0)	29 (2.0)	– 1.1% (– 2.1 to 0.0%)
Ataxia^a^	76 (3.6)	37 (2.5)	– 1.1% (– 2.2 to 0.1%)
Any neurological deficit^a^	183 (8.6)	97 (6.5)	– 2.1 (– 3.8 to 0.3%)

*SD* standard deviation, *CI* confidence interval, *TIA* transient ischemic attack, *AVS* acute vestibular syndrome

^a^Several categories of findings were present in the same patient in *n* = 33 subjects during period 1, and *n* = 13 patients during period 2

### Effectiveness markers and diagnoses

The pre-defined effectiveness markers are presented in Tables 2 (outcomes a–e) and 3 (outcome f). The proportion of symptom diagnoses did not change (Table 2, outcome a), however, the proportion of the study population undergoing Dix–Hallpike testing increased during period 2 (Table 2, outcome b). This was mirrored by an increase in the proportion diagnosed with BPPV, from 7.6% (95% CI 6.6–8.8%) in period 1 to 15.3% (95% CI 13.6–17.3%) in period 2. The increase in BPPV diagnoses was explained by smaller shifts from several other categories. Removing the *n* = 11 patients in period 2 who had their unspecific diagnoses changed to BPPV as detailed in the methods section did not affect this finding, 7.6% (95% CI 6.6–8.8%) vs. 14.5% (95% CI 12.8–16.4). The proportion diagnosed with stroke did not differ between the periods, 2.9% (95% CI 2.3–3.7%) in period 1 vs. 2.8% (95% CI 2.1–3.8%) in period 2. Furthermore, after the implementation of the algorithm, a lower proportion of ED visits led to hospitalization and the number of days spent in-hospital decreased (Table 2, outcome c). Patients without a medical diagnosis had the largest reduction in proportion admitted, 54.1% (95% CI 51.2–56.9%) in period 1 vs. 34.5% (95% CI 31.2–37.9%) in period 2. The reductions in proportion admitted and length-of-stay were mirrored by a small but non-significant decrease in the number of days spent in-hospital by the patients in the control group with headache, median 2 vs. 2, mean 2.44 vs. 2.11, *n* = 1213, *p* = 0.30 (Mann–Whitney *U* test). During the course of the study the mean number of available hospital beds at the wards (stroke, medicine and neurology) caring for the majority of the admitted dizzy patients 1781/2016 [88.3% (95% CI 86.9–89.7%)], decreased from 105.2 during period 1 to 84.1 during period 2. However, over the years 2014–2017 (data not available for 2012 and 2013), the ward occupancy increased by 0.8% yearly from 95.3 to 98.5%. Fewer patients underwent a neuroradiological investigation after the implementation of the algorithm with a shift from CTs to MRIs (Table 2, outcome d). Despite this, the diagnostic yield of the MRIs remained unchanged while the diagnostic yield of CTs doubled. The most common neuroradiological findings over the 5-year study duration were ischemias (*n* = 64), tumors (*n* = 16), intraparenchymal hematomas (*n* = 14), subdural hematomas (*n* = 12) and inflammatory lesions (*n* = 4). There were no differences between the proportion of patients returning with a new dizzy spell within 90 days, or the proportion of dizzy patients remaining stroke-free over the 90 days following their ED visit during the periods (Table 2, outcome e). The costs per patient declined after the implementation of the management algorithm, from $2561 in period 1 to $1808 in period 2 (Table 3, outcome f).

**Table 2:** Pre-defined effectiveness markers (a–e) for the care of dizzy patients at Umeå University Hospital comparing period 1 (2012–2014) and period 2 (2016–2017)

Outcome	Period 1 (*n* = 2126)	Period 2 (*n* = 1487)	Difference (95% CI)
(a) Symptom diagnoses, *n* (% of total)	1174 (55.2)	796 (53.5)	– 1.7% (– 5.0–1.6%)
(b) Vestibular testing			
Dix–Hallpike tested, *n* (% of total)	299/1437 (20.8)^a^	560 (37.7)	16.9% (13.6 to 20.1%)
Referred to ENT, *n* (% of total)	322 (15.2)	279 (18.8)	3.6% (1.1 to 6.2%)
(c) Admittance			
Hospitalization, *n* (% of total)	1308 (61.5)	708 (47.6)	– 13.9% (– 10.6 to – 17.2%)
Days spent in hospital, mean (SD)^b^	3.85 (3.6)	3.02 (3.4)	– 0.83 (– 1.15 to – 0.51)
(d) Neuroradiology			
CT, *n* (% of total)	891 (41.9)	382 (25.7)	– 16.2% (– 19.2 to – 13.1%)
MRI, *n* (% of total)	209 (9.8)	248 (16.7)	6.9% (4.6–9.2%)
Any neuroradiology, *n* (% of total)	953 (44.8)	539 (36.3)	– 8.6% (– 11.8 to – 5.3%)
Patients undergoing both CT and MRI, *n* (% of total)	147 (6.9)	91 (6.1)	– 0.8% (– 2.4–0.9%)
Diagnostic MRI,* n* (% of total)^c^	30/209 (14.4)	34/248 (13.7)	– 0.6% (– 7.2–5.7%)
Diagnostic CT, *n* (% of total)^c^	43/891 (4.8)	36/382 (9.4)	4.6% (1.6–8.2%)
(e) 90-day outcomes			
ED Dizziness return visit within 90 days, *n* (% of total)^d^	151 (8.1)	83 (6.8)	– 1.3% (– 3.2–0.6%)
Stroke-free within 90 days, *n* (% of total)^e^	2119 (99.7)	1480 (99.5)	– 0.1 (– 0.7–0.3)

^a^Data from 2012 to 2013 only, see Methods

^b^The median (interquartile range) days spent in hospital were 2 (1–3) and 3 (2–4), for period 1 and 2, respectively,* p* < 0.01 (Mann–Whitney *U* test)

*CI* confidence interval, *ENT* ear–nose–throat, *SD* standard deviation, *CT* computed tomography, *MRI* magnetic resonance imaging, *ED* emergency department

^c^CTs or MRIs with findings with a plausible association with the dizziness

^d^Only index visits during 2012-01-01 until 2014-09-30 for period 1 (*n* = 1861), and 2016–01-01 until 2017–09-30 for period 2 (*n* = 1222) were considered to allow for a full 90 day follow-up after all visits

^e^The number of patients without a cerebrovascular diagnosis at index who remained stroke-free were similar, 99.7% (2017/2024) vs. 99.6% (1423/1429) during period 1 vs. period 2

**Table 3:** Total, yearly and per-visit expenses in US dollars for dizzy patients at Umeå University Hospital comparing period 1 (2012–2014) and period 2 (2016–2017)

Category	Period 1 (*n* = 2126)	Period 2 (*n* = 1487)
Emergency department visits^a^	$1,042,131	$728,904
Neuroradiology		
CT^b^ (total no. of scans)	$320,751 (891)	$137,516 (382)
MRI^c^ (total no. of scans)	$111,251 (209)	$132,011 (248)
Total radiology	$432,002	$269,527
Yearly radiology	$144,001	$134,763
Radiology per visit	$203	$181
Radiology per diagnostic scan^d^	$5918	$3850
Hospital care^e^		
Total costs for days in hospital (no. of days)	$3,970,432 (5041)	$1,690,249 (2146)
Yearly costs	$1,985,216	$845,125
Costs per visit	$1868	$1137
Total costs		
Per year	$1,814,855	$1,344,340
Per visit	$2561	$1808

All calculations are based on the 2017 tariff. The exchange rate used to convert Swedish currency (SEK) to US currency (US dollars) was 1 US dollar = 9.78 SEK (8th September 2018)

*US* United States, *CT* computed tomography, *MRI* magnetic resonance imaging, *ED* emergency department

^a^One visit at the emergency department including all costs related to administration, equipment, personell, facilities and laboratory analyses (but not radiology) costed $490 during 2017 (a mean calculated for all ED patients)

^b^CT scan costs for an acute investigation (base price without angiogram $188, with angiogram $407) were weighted based on 29.7% angiograms and the following distribution over the days and weeks: 40% weekdays 07.30–16.30 (base + $6), 23.1% weekdays 16.31–21.00 (base + $31), 7.7% weekdays 21.01–00.00 (base*2), and 29.2% weekdays 00.01–07.29 and weekends/holidays (base*2). Mean cost per CT scan was thus approximated to $360

^c^The cost for one MRI was $532

^d^A CT scan or an MRI was considered diagnostic when revealing finding(s) with a plausible association with the presenting symptoms. No. of diagnostic scans during period 1 *n* = 73, period 2 *n* = 70. When both CT and MRI were performed and revealed relevant findings, both were considered diagnostic

^e^Mean cost per day at the hospital wards (stroke, neurology, internal medicine) where the majority of dizzy patients are treated was $788

## Discussion

This single-center interventional study has shown that the implementation of a comprehensive management algorithm for dizzy patients at Umeå University Hospital was associated with improved effectiveness of care for this patient group. The proportion of patients undergoing Dix–Hallpike testing as well as receiving a BPPV diagnosis doubled, the number of days spent in-hospital decreased, and the proportion of patients undergoing neuroradiological investigations decreased without an increased number of missed strokes. Taken together, these changes have led to lower costs for the care of these patients. As earlier studies have suggested high and rising costs for neuroradiology among dizzy patients, and very low proportions undergoing Dix–Hallpike testing (3.9%) and receiving a BPPV diagnosis (4.4%), we believe that our observations are important also outside the Swedish setting [6, 13].

### Effects on diagnoses (outcomes a and b)

The proportion undergoing Dix–Hallpike testing increased after the implementation of the management algorithm and this probably stems directly from the instructions and the educational efforts with nurses and physicians (outcome b). As a result, the rate of BPPV diagnoses almost doubled. Despite this and other efforts to increase diagnostic accuracy the number of patients receiving non-specific symptom diagnoses remained high compared with earlier studies (outcome a) [3, 8]. The reasons for this are unclear. There were several small shifts between the proportions of symptom diagnoses and various medical diagnoses without a clear pattern (data not shown), overall preventing a reduction of the proportion undiagnosed. Perhaps a longer study duration with more focus on diagnoses outside of the neurological and neurotological spheres could have been more effective to push practice away from using symptom diagnoses.

### Effects on management, follow-up and costs (outcomes c–f)

A lower proportion of patients were admitted, and the length-of-stay decreased after the implementation of the management algorithm (outcome c). The largest reduction in admission was seen for patients with non-specific symptom diagnoses. The reasons behind this are not readily deducted by our data, however, we may speculate that less diagnostic uncertainty at the ED contributed to fewer admissions of patients with a low risk for dangerous causes of dizziness. Possible reasons for the shorter duration of in-hospital stays include better diagnostic procedures and the benefits of a step-wise algorithm for AVS patients which standardizes management. It is also reasonable to assume that the decrease in available hospital beds during the study influenced these data, to some extent supported by the observation that undiagnosed patients were more seldom admitted, i.e. patients with the supposed lowest medical priority. However, our analyses of headache patients, a control group without management changes during the study, suggested that the overall decrease in length-of-stay at these wards was not as pronounced as the decrease observed among dizzy patients. Also, the increased occupancy over the years mitigates, to some extent, the decreased availability of beds.

The shift from CT to MRI was an inherent part of the management algorithm (outcome d). This shift is appropriate as the sensitivity for detecting stroke is higher with MRI, 83% for MRI vs. 26% for CT for the diagnosis of any stroke, imaged between 36 min and 8 days after symptom onset [14, 15]. It is reassuring to notice that despite a lower total use of neuroradiology, and lower costs for neuroradiological investigations, the rate of new onset stroke within 90 days did not increase but stayed on levels comparable with previous reports [16]. This suggests that the sensitivity for detecting stroke did not decrease (outcome e). Also, as shown in Table 2, the more selective use of CT during period 2 was associated with a higher diagnostic yield than in period 1. We expected a lower proportion of patients returning with a new dizzy spell within 90 days during period 2 due to improved ability to detect and cure BPPV (outcome e), however, we did not detect such an effect. This may be due to a lack of such an effect, due to low power, or perhaps due to inadequate discharge information delivered to patients with BPPV.

The decreases in admittance and neuroradiology use were, naturally, mirrored by lower costs for patient care (outcome f). It is difficult to calculate the costs of care in a publicly financed health care system with minimal patient fees (i.e. $20 for an ED visit, $10/day for in-patient care). For the calculations in this paper we used the organization’s economic governance groups’ pricing lists, as detailed in the methods. To actually lead to lower expenses, reductions in utilization of resources must invoke reductions in availability. It is possible that rather than savings, the reductions in utilization of these resources by dizzy patients has led to higher availability for other patient categories.

### Baseline data comparisons

When comparing the two periods, most baseline characteristics and comorbidities were well matched. The exceptions were the rates of hyperlipidemia, congestive heart failure and previous stroke, in effect making the cohort in period 1 slightly less healthy. These differences in baseline characteristics might have influenced the outcomes to a small degree but it is unlikely that they can explain all the differences seen. Fewer patients fulfilled the AVS criteria in period 2, perhaps due to a more comprehensive exclusion of BPPV patients. Furthermore, a larger proportion of AVS cases had nystagmus, likely reflecting the active search for this sign and more targeted documentation.

### Limitations

Limitations of this study include the retrospective data collection, separate periods for data collection, and the time-period comparison design making it vulnerable to missing or undocumented data, data abstractor bias and regression to the mean. Unfortunately, the data source did not allow for blinding of selected data such as date. Efforts to mitigate this were taken through uniform data collection instructions over the periods, strict adherence to protocol-defined diagnoses and attempts to control for non-related organizational changes over time. Although this is somewhat problematic, missing data were considered not present, as the retrospective data collection rules out the possibility to direct physicians’ documentation for data completeness. That some of the diagnoses were re-evaluated and changed retrospectively is also problematic as all personnel working with the data were unblinded. An effort to overcome this with regards to the changes in the proportions of BPPV diagnoses by removing retrospectively assigned BPPV diagnoses during period 2 did not suggest that such errors influenced the analyses. Another shortcoming is that all aspects of care were not included in the health economy evaluations, most notably the in-patient laboratory costs (which were unfortunately not available); and that the costs for ED visits and in-patient care were not calculated on individual level but as a per unit average.

## Conclusions

In conclusion, this study suggests that the implementation of a management algorithm for dizzy patients at Umeå University Hospital was associated with an increased effectiveness of care for these patients. Further research is warranted to better understand why many patients remain undiagnosed despite increased efforts to find the causes of dizziness, and to develop optimal decision trees for stroke risk stratification.
